# Does adoption of the rice–crayfish co-culture model improve farmers’ income?

**DOI:** 10.1371/journal.pone.0328292

**Published:** 2025-09-11

**Authors:** Xingjie Yang, Zhenhong Qi

**Affiliations:** 1 College of Economics and Management, Zhejiang A & F University, Linan, Zhejinag, China; 2 College of Economics and Management, Huazhong Agricultural University, Wuhan, Hubei, China; Bangladesh Agricultural University, BANGLADESH

## Abstract

This study empirically assesses the income effects of the rice–crayfish co-culture model using endogenous switching regression (ESR) and mediation models, based on survey data from 1,058 farm households in Hubei Province. Key findings reveal that adoption of the rice–crayfish co-culture model significantly boosts farmers’ total income: counterfactual analysis shows non-adopters would experience a 22.423% decline in average household income if they ceased adoption. The adoption of the rice–crayfish co-culture model differential impacts on the income of various farmer groups. This divergence primarily stems from the model’s significantly stronger positive effect on agricultural income compared to its minimal dampening effect on non-farm income. By examining the mechanism of its effect on farmers’ income, we find that adopting the rice–crayfish co-culture model mainly promotes farmers’ income by affecting the human capital of the family. Based on the above conclusions, the Chinese government should further promote the sustainable development of the rice–crayfish co-culture model and give full play to its role in increasing farmers’ income. Simultaneously, constructing a comprehensive industrial system for the rice–crayfish co-culture model and intensifying technical training are imperative. These efforts aim to enhance the human capital of farmers, which in turn will effectively promote the growth of their income.

## Introduction

Income has been discussed as a significant problem for developing countries. Specifically, low income is a threat to farmers’ livelihood in China, if farmers’ income problem is not effectively solved, it will not only affect the sustainable development of the rural economy but also hinder the realisation of the strategic goals of rural revitalisation [1,2]. How to solve this problem has become the focus of attention of people from all walks of life.

The extant scholarship on the determinants of farmers’ income has predominantly been classified along two analytical dimensions: internal and external factors. Internal determinants, as elucidated in seminal works by Islam [3], Emongor and Uside [4], Goeb and Lupi [5], and Jin [6], encapsulate variables including educational levels, vocational skill development, access to irrigation infrastructure, and individual health conditions. Conversely, external determinants, as underscored by research conducted by Guillaume and Paule [7], Alizamir et al.[8], Biagini et al.[9], and Goel et al.[10], emphasize the criticality of digital agricultural production systems, cooperative membership, governmental policy architectures, and contract farming mechanisms. Collectively, these academic explorations demonstrate that increasing farmers’ income is a multifaceted function, profoundly shaped by the intricate interplay between internal endowments and external supportive conditions.

With the continuous deepening of academic research, scholars have increasingly focused on the rice–crayfish co-culture model. Grounded in the theoretical paradigms of biological symbiosis and resource optimization, this agro-ecological system has emerged as a pivotal strategy for strengthening rural livelihood resilience [11]. The underlying mechanism of this agro-ecological system is predicated on the multifunctional role of procambarus clarkii within paddy ecosystems. As opportunistic omnivores, crayfish actively consume aquatic weeds, insect pests, and decomposing organic matter, thereby reducing reliance on synthetic pesticides. Their excretions, rich in nitrogen, phosphorus, and potassium, functions as an endogenous nutrient source, facilitating optimal rice growth through natural fertilization [12,13]. Recognized as a major agricultural innovation by China’s Ministry of Agriculture, the rice–crayfish co-culture system has been extensively promoted across multiple provinces. Notably, regions in the Middle and Lower Reaches of the Yangtze River Plain, characterized by favorable hydrological and topographical conditions, have witnessed the highest adoption rates and most substantial yield enhancements.

Nevertheless, the ric–crayfish co-culture model still has some constraints in its practical application. First, the input cost of reconstructing the paddy field infrastructure is high, and the investment can only be gradually recovered in later operation, that is, there is a certain time difference between the time of investment and return, which increases the uncertainty of the investment returns. Second, the rice–crayfish co-culture model has a specific technical threshold, and farmers often find it more difficult to increase their income in production due to insufficient technical experience [14,15]. These challenges prompt several critical research questions: Does the adoption of the rice–crayfish co-culture model increase farmers’ incomes? If so, what are the mechanisms through which it increases farmers’ income? Additionally, how does the impact of this model vary across different farmer demographics? Answering the above questions has important theoretical and practical significance for promoting the sustainable development of ecological agriculture model and increasing farmers’ enthusiasm for adopting.

Focusing on the income effect of farmers’ adoption behavior, some scholars argue that, on the one hand, as people’s consumption demand for green agricultural products increases, the market premium of agricultural products produced through ecological and green prevention and control is strong, enabling farmers to increase their income [16–18]. On the other hand, the new agricultural technology model has certain complexity and systematicness. In the early stage of technology adoption, as the input–output benefit ratio is low, it is difficult to achieve the goal of increasing income by adopting new technologies [19,20]. It is worth noting that the rice–crayfish co-culture model is different from farmers adopting a certain technology. This is because the rice–crayfish co-culture model organically integrates planting and aquaculture technologies, making it more systematic and holistic. Once farmers adopt this model, it will run through the whole process of agricultural production, and the income-increasing effect after adoption also mainly depends on whether its management is standardised.

Can the rice–crayfish co-culture model effectively drive farmer income growth? Previous research, including studies by Islam et al.[21] and Chen et al.[22], has employed econometric models to investigate the income implications of this innovative cultivation approach. The rice–crayfish co-culture model prioritizes high quality, ecological sustainability, and food safety [23]. Grounded in the principles of coordination, circularity, and long-term viability, it drives agricultural transformation through technological advancements. The rice–crayfish co-culture model has been proven to significantly enhance paddy field productivity, as documented by Si et al.[24] and Zhu et al.[25]. Furthermore, the rice–crayfish co-culture model ingeniously integrates rice cultivation with crayfish farming, creating a “one field, dual use” system [26]. This synergy maximizes paddy field resource utilization. Through harnessing the symbiotic relationship between rice and crayfish, the model reduces the reliance on chemical fertilizers and pesticides, thereby ensuring product quality. Findings from research by He et al.[27] and Qiao et al.[28] indicate that, compared to monoculture rice farming, the rice–crayfish co-culture model imposes less environmental stress, particularly in terms of ecosystem disruption. Notably, from the perspective of continuous adoption, existing research has found that farmers’ continuous adoption of the rice–crayfish co-culture model can increase their net agricultural income by 83,430 yuan [29]. However, the research perspective and mechanism of action still need to be deepened to find the impact mechanism of the rice–crayfish co-culture model on farmers’ income.

The contributions of this study can be summarized as follows: First, the research centers on the rice–crayfish co-culture model, which is mainly popularised and applied in the Jianghan Plain. By employing mathematical derivations and empirical analyses, we construct a counterfactual framework to test whether and the extent that the adoption of the rice–crayfish co-culture model help farmers increase their income. Through this approach, the income-increasing effect of the rice–crayfish co-culture model is comprehensively investigated, and a scientific basis is provided for the government to formulate a promotion plan. Second, the study delves deeper into the differential impacts of the rice–crayfish co-culture model on agricultural and non-agricultural income streams. The findings shed light on whether the model exerts varying effects across different components of farmers’ income portfolios. Third, through the application of a mediation effect model, this research elucidates the underlying mechanisms by which the rice–crayfish co-culture model drives income growth for farmers. These insights offer a nuanced understanding of the model’s contribution to enhancing farmers’ economic well-being.

## Theoretical analysis and empirical model

### The direct action mechanism of the adoption of the rice–crayfish co-culture model to increase farmers’ income

The rice–crayfish co-culture model is based on the theory of two types of agriculture—organically combining planting and breeding technologies. It not only coordinates the relationship between rice and crayfish cultivation in the same field but also realises the recycling of resources between the two, providing an innovative solution for the transformation of the traditional agricultural development model to modern agriculture, which has an impact on farmers’ income. To analyse the direct action mechanism of adopting the rice–crayfish co-culture model to increase farmers’ income, following the existing research [30], we construct the following production function model:


$$Q=F(K,AL)={K(t)}^{\alpha }{\left[A(t)L(t)\right]}^{1-\alpha }$$ (1)

In Equation (1), Q\  is the total output of rice and crayfish production; K(t) is capital investment, such as digging crayfish trenches and purchasing anti-escape nets. A(t) is technological progress; L(t) is labour input; α and \ 1−α are the elasticity of capital and labour, respectively, and α is between 0 and 1.

Assuming the returns to scale for farmers adopting the rice–crayfish co-culture model remain unchanged, then:


$$Y(t)=A(t)L(t){\left[\frac{K(t)}{A(t)L(t)}\right]}^{\alpha }=A(t)L(t)f(k)$$ (2)

where $y=f(k)=k^{\alpha }={\left[\frac{K(t)}{A(t)L(t)}\right]}^{\alpha }$; y is the average output per unit of agricultural labour, and k is the average capital per unit of agricultural labour. By taking the partial derivative of capital and labour inputs, the marginal product of capital and labour can be obtained as follows:


$${MP}_{K}=\frac{\partial \left\{{K(t)}^{\alpha }{\left[A(t)L(t)\right]}^{1-\alpha }\right\}}{\partial K}=f^{\prime }(K)$$ (3)

$${MP}_{L}=A(t)\left[f(K)-Kf^{\prime }(K)\right]$$ (4)

According to Equation (4), the marginal product of labour is mainly affected by technological progress A(t), the average output of labour L(t), and the average output of capital per unit labour $Kf^{\prime }(K)$, $\;A(t)>0\text{, }f(K)-Kf^{\prime }(K)>0$.

In addition, assuming that the market price of agricultural products produced by farmers adopting the rice–crayfish co-culture model is *P*, the income $I_{A}$ that farmers can obtain by adopting the rice–crayfish co-culture model is as follows:


$$I_{A}=P*Q=P*{MP}_{L}=P*A(t)[f(K)-Kf^{\prime }(K)]$$ (5)

From Equation (5), the income effect of farmers’ adoption of the rice–crayfish co-culture model is affected by the price of rice and crayfish products P, technological progress A(t), the combined effect of the average output of labour f(K) and the output of capital $Kf^{\prime }(K)$ per unit of labour. This is because A(t)>0, and $f(K)-Kf^{\prime }(K)>0$. Therefore, when the marginal product of labour ${MP}_{L}>0$, the income $I_{A}$of adopting the rice–crayfish co-culture model is positive. The above analysis reveals that the rice–crayfish co-culture model is a new production technology, and farmers can achieve income growth by adopting this model.

Nevertheless, the direct action mechanism of adopting the rice–crayfish co-culture model to increase farmers’ income needs to be further refined, because the income-increasing effect of the rice–crayfish co-culture model is closely related to the standardisation of farmers’ management. After farmers adopt it, there are two possibilities for extensive and refined management. Assuming that they are “rational small farmers”, they will adopt rice–crayfish co-culture model mainly to maximise their income. Therefore, they will choose to invest a certain amount of time and energy in refined management rather than extensive management, which has higher requirements for the allocation of labour resources. As the labour resources of farm households are limited, coupled with the scarcity of labour time, the resource allocation between agricultural and non-agricultural peasant households has a trade-off relationship [31]. Farmers need to put more labour resources into agricultural production when adopting the rice–crayfish co-culture model, increase farm income through refined management and reduce the time and labour used for off-farm production. Therefore, the income-increasing effect of adopting the rice–crayfish co-culture model is mainly from farm income. This leads to the first hypothesis (H1):

H1: On the premise of controlling other factors, the total income of farmers adopting the rice–crayfish co-culture model is significantly higher than that of farmers not adopting the model, and the income-increasing effect mainly comes from the growth of agricultural income.

### The indirect mechanism of the adoption of the rice–crayfish co-culture model to increase farmers’ income

The indirect effect mechanism of adopting the rice–crayfish co-culture model on farmers’ income is mainly reflected in the following aspects: the popularisation and application of the rice–crayfish co-culture model help improve the level of rural human capital, which is embodied in two aspects–labour quantity and quality. Moreover, improving rural human capital has an important impact on promoting farmers’ income [32].

First, the impact of adopting the rice–crayfish co-culture model on human capital is mainly reflected in two aspects. On the one hand, the rice–crayfish co-culture model involves the coordinated symbiosis of rice planting and crayfish breeding, thereby prolonging the agricultural production link and process. To ensure the organic connection of the entire production process, a certain amount of labour is required as support. Therefore, the promotion and application of the rice–crayfish co-culture model will help drive more rural labours to engage in agricultural production, thereby increasing the number of agricultural labours. On the other hand, compared with traditional agricultural production methods, the rice–crayfish co-culture model effectively improves the production efficiency of paddy fields and protects the interests of agricultural operators [33], thereby increasing the income of farmers. As some farmers with high education level and rich production experience are rooted in rural areas and are engaged in agricultural production and operation, this will improve the quality of rural agricultural labour and ultimately improve the level of human capital in the entire rural area through spillover effect.

Second, improving the level of human capital also helps to increase the income of farmers [34]. According to the new economic growth theory, the main driving force of the transformation and upgrading of agricultural production comes from improving production efficiency, and human capital is the key factor that affects the improvement of production efficiency [35]. This is because improving human capital directly increases farmers’ income by improving resource utilisation efficiency and agricultural production efficiency. This is reflected in two aspects. On the one hand, improving the level of human capital would not only make agricultural producers more aware and capable of specialising in production but also helps to guide agricultural producers to divide labour according to market requirements so that they would be in a state of high efficiency throughout the production process [36]. On the other hand, in the context of agricultural transformation and upgrading, agricultural development models and production equipment are constantly innovating, which objectively requires that the quality of labours should be effectively matched with continuous improvement. The adoption of the rice–crayfish co-culture model to improve human capital levels is endogenously consistent with the new requirements of the transformation and upgrading of agriculture to improve agricultural marginal productivity and ultimately increase the income of farmers. Based on this, the second hypothesis (H2) is proposed:

H2: On the premise of controlling other variables, the adoption of the rice–crayfish co-culture model will indirectly promote income growth by enhancing farmers’ human capital.

### Empirical model

Endogenous switching regression model (ESR). As farmers’ adoption of the rice–crayfish co-culture model is impacted by the problem of self-selection caused by the external environment and their own characteristics and there are some unobservable factors that affect the adoption behaviour and income simultaneously, if regression analysis is not considered, the results are likely to be biased. Referring to the ESR proposed by Maddala [37], first, the model considers the influence of both observable and unobservable factors. Second, the model estimates the factors affecting the income of the adopting and non-adopting groups simultaneously. Third, calculate the counterfactual estimations. The model mainly includes two stages. The first stage uses the probit model to estimate the adoption behaviour equation of the rice–crayfish co-culture model, and in the second stage, the income equation is established to estimate the impact of adopting the rice–crayfish co-culture model on farmers’ income.

According to the expected utility theory, assuming that farmers adopt the rice–crayfish co-culture model to obtain potential net income $A_{ia}^{*}$, the potential net benefit for non-adopters is $A_{in}^{*}$, and the conditions for the farmers to adopt is $A_{ia}^{*}-A_{in}^{*}=A_{i}^{*}>0$. Although $A_{i}^{*}$ cannot be directly observed, it can be represented by an observable exogenous variable. Thus, $A_{i}$ can be expressed as follows:


$$A_{i}=\{\begin{array}{cc} 1, & A_{i}^{*}>0\\ 0, & A_{i}^{*}\leq 0 \end{array}\}$$ (6)

In Equation (6), $A_{i}$ denotes the adoption behaviour of farmers, where $A_{i}=1$ indicates that farmers have adopted the rice–crayfish co-culture model, and ${\;A}_{i}=0$ means that it has not been adopted. The model for assessing the impact of adopting the rice–crayfish co-culture model on farmers’ income is as follows:


$$Y_{i}=\beta X_{i}+\gamma A_{i}+\varepsilon_{i}$$ (7)

In Equation (7), $Y_{i}$ represents the total income; $X_{i}$ represents factors that influence income; β and γ are the parameters to be estimated, and $\varepsilon_{i}$ is a random disturbance term. The specific equation is as follows:

Behaviour equation (whether to adopt the rice–crayfish co-culture model):


$$A_{i}=\delta Z_{i}+KI_{i}+\mu_{i}$$ (8)

Solving Equation 6 (the total income of farmers that adopt the rice–crayfish co-culture model is the treatment group), we derive the following:


$$Y_{ia}=\beta_{a}X_{ia}+\varepsilon_{ia}$$ (8a)

Solving Equation 7 (the total income of farmers that do not adopt the rice–crayfish co-culture model is the control group), we derive the following:


$$Y_{in}=\beta_{n}X_{in}+\varepsilon_{in}$$ (8b)

In Equation (8), $Z_{i}$ is the factor that affects the adoption behaviour; $\mu_{i}$ is a random disturbance term, and $I_{i}$ is an instrumental variable. In equations (8a) and (8b), $Y_{ia}$ and $Y_{in}$ are the total revenue of the treatment and control groups, respectively; $X_{ia}$ and $X_{in}$ are the influencing factors, and $\varepsilon_{ia}$ and $\varepsilon_{in}$ are random disturbance terms.

To estimate the impact of adopting the rice–crayfish co-culture model on farmers’ income, it is necessary to use the counterfactual analytical framework to compare the expected value of the income level under the real and counterfactual scenarios and then estimate the average treatment effect (ATT) of adopting the rice–crayfish co-culture model on the income of farmers.

The expected income of the treatment group is as follows:


$$E[Y_{ia}\;|\;A_{i}=1]=\beta_{a}x_{ia}+\sigma_{\mu a}\lambda_{ia}\;$$ (9)

The expected income of the control group is as follows:


$$E[Y_{in}\;|\;A_{i}=0]=\beta_{n}x_{in}+\sigma_{\mu n}\lambda_{in}\;$$ (10)

The expected income when the treatment group does not adopt is as follows:


$$E[Y_{in}\;|\;A_{i}=1]=\beta_{n}x_{ia}+\sigma_{\mu n}\lambda_{ia}\;$$ (11)

The expected income when the control group adopts is as follows:


$$E[Y_{ia}\;|\;A_{i}=0]=\beta_{a}x_{in}+\sigma_{\mu a}\lambda_{in}\;$$ (12)

Therefore, the actual average treatment effect of adopting the rice–crayfish co-culture model (ATT) is expressed as the difference between Equations (9) and (11):


$${ATT}_{i}=E[Y_{ia}\;|\;A_{i}=1]-\;E[Y_{in}\;|\;A_{i}=1]=\left(\beta_{a}-\beta_{n}\right)x_{ia}+\left(\sigma_{\mu a}-\sigma_{\mu n}\right)\lambda_{ia}\;$$ (13)

Conversely, the average treatment effect of not adopting the rice–crayfish co-culture model (ATU) is expressed as the difference between Equations (10) and (12):


$${ATU}_{i}=E[Y_{in}\;|\;A_{i}=0]-\;E[Y_{ia}\;|\;A_{i}=0]=\left(\beta_{n}-\beta_{a}\right)x_{in}+\left(\sigma_{\mu n}-\sigma_{\mu a}\right)\lambda_{in}\;$$ (14)

In conclusion, the average treatment effect of adopting the rice–crayfish co-culture model on farmers’ income is the average of ${ATT}_{i}$ and ${ATU}_{i}$.

Mediating effect model. To examine the mechanism of adopting the rice–crayfish co-culture model on the total income of farmers, by referring to the existing research [38], we construct the mediation effect test steps for empirical analysis. The specific model is as follows:

Step 1: Analyse the total effect of adopting the rice–crayfish co-culture model on farmers’ income:


$$\begin{array}{c} Y_{i}=\alpha_{0}+\alpha_{1}A_{i}+\sum {\mathrm{\backslash nolimits}}_{n=1}^{N}\alpha_{i}X_{i}+\tau_{1} \end{array}$$ (15)

Step 2: Analyse the impact of adopting the rice–crayfish co-culture model on the mediating variable:


$$\begin{array}{c} F_{i}=\pi_{0}+\pi_{1}A_{i}+\sum {\mathrm{\backslash nolimits}}_{n=1}^{N}\pi_{i}X_{i}+\tau_{2} \end{array}$$ (16)

Step 3: Analyse the impact mechanism of the mediator variable on the income of farmers in the adoption behaviour:


$$\begin{array}{c} Y_{i}=\theta_{0}+\theta_{1}A_{i}+\theta_{2}F_{i}+\sum {\mathrm{\backslash nolimits}}_{n=1}^{N}\theta_{i}X_{i}+\tau_{3} \end{array}$$ (17)

In Equations (15) to (17), $Y_{i}$ is the total income of the farm household; $A_{i}$ is the adoption behaviour of farmers; $X_{i}$ is other influencing factors; $F_{i}$ is the mediating variable; this article mainly refers to the human capital; $\tau_{i}$ is a random disturbance term.

## Data sources and research design

### Data sources

The data in this study were from the field survey of farmers households in Hubei province in the Jianghan Plain from August to September 2021. The following factors were considered before selecting the Jianghan Plain. First, Jianghan Plain has superior water and arable land resources and is the main promotion area for the rice–crayfish co-culture model. Second, Jianghan Plain is located in the middle reaches of the Yangtze River, with densely distributed water networks and lakes. It has a subtropical climate and is suitable for both rice and crayfish cultivation. Third, the rice–crayfish co-culture model in the Jianghan Plain has developed rapidly, among which Hubei Province ranks first in the country in terms of planting area and output (http://www.shuichan.cc/news_view-403596.html), and Hubei Province has innovative units, such as the Institute of Hydrobiology of the Chinese Academy of Sciences and the Yangtze River Fisheries Research Institute of the Chinese Academy of Fishery Sciences. The scientific research strength and research level are relatively high, which provides an important guarantee for the standardised development of the rice–crayfish co-culture model. So, choosing the Hubei Province as the research area has certain representativeness.

Before the formal survey, the research group conducted a preliminary survey in Shashi District, Hubei Province, and revised the questionnaire according to the survey results. The content of the investigation includes the basic situation of farmers, farmers’ adoption of the rice–crayfish co-culture model and the input-output situation of agriculture. The stratified random sampling method was employed in the survey. The specific steps are as follows. First, we comprehensively considered the overall layout of the rice–crayfish co-culture model “two belts, three circles and four districts” (‘Two belts’ refer to the ‘double water and double green’ industrial belt of the Yangtze River and the ‘double water and double green’ industrial belt of the Han River; ‘three circles’ refer to the characteristic development circle of ‘double water and double green’ in central Hubei, and the ‘double water and double green’ of Jianghan Plain. The key development circle is the modelrate development circle of ‘double water and double green’ in eastern Hubei; ‘four districts’ refer to the core area, the advantageous area, the suitable area and the sub-suitable area). The survey mainly focused on nine counties in the key development circle of “Double Water and Double Green” in the Jianghan Plain, covering Jingzhou District, Shashi District, Jiangling County, Gongan County, Shishou District, Jianli County, Honghu District, Xiantao District and Qianjiang District. Second, in the nine counties, according to the standard that the area of the rice–crayfish co-culture model accounts for 80%, 60%, 40% and 20% of the total cultivated land in the county, a township is selected, selecting 36 townships in total. Moreover, each township randomly chooses 2 villages, selecting a total of 72 villages. Finally, in the selected villages, 15 farmers were randomly extracts as the final survey object, so a total of 1,079 questionnaires were distributed.

### Variable definition and descriptive statistics

According to the model setup, the dependent variables of the endogenous switching regression model are adoption behaviour and income level. Following existing research [39], we set adoption behaviour as a binary variable. Regarding the income level, scholars primarily use living consumption expenditure, household economic income, and other variables to measure it [40]. Due to significant differences among farm households, we use more comparable per capita net income as the core dependent variable, the ratio of a household’s annual net income to the total number of people. Furthermore, based on the logic of decomposing household annual net income from previous studies [41], two additional dependent variables were obtained: farm income and off-farm income.

Existing research primarily selects five types of variables as independent variables: individual, family, operational, other, and village characteristics [39,42]. Individual characteristics include gender, age, and health status. Family characteristics include the ratio of elderly individuals, relationship networks, and farming years. Operational characteristics include cultivated land area, soil fertility, the number of arable lands, and farmland distance. Other characteristics include ecological consciousness and loan situations. Village characteristics include village terrain, village irrigation, and distance from town.

To make the model identifiable, instrumental variables need to be introduced into the selection equation. Previous studies have suggested that peer effects are widespread in rural areas, meaning that farmers’ behavioral decisions are influenced by those of other farmers in the same village [43]. For instance, scholars who randomly assigned university students to different groups discovered that peer effects could effectively enhance interaction among group members [44].

Based on this, the paper establishes an instrumental variable for farmers’ adoption behavior, using the peer group effect identified in existing relevant research. Specifically, the mean behavior of farmers who adopt the rice–crayfish co-culture model is used as the instrumental variable in relation to other villagers [45]. The primary rationale for this choice is that, from a social learning perspective, farmers in rural areas exchange information with their neighbors during production and business activities. These social learning effects prompt other farmers to change their decision-making behavior and adopt the same decision-making behavior as their neighbors [46]. Following this logic, once neighboring farmers adopt the rice–crayfish co-culture model, social learning will prompt other farmers to do the same, thereby satisfying the relevance principle of the instrumental variable. Nevertheless, the adoption behavior of other farmers in the village belongs to the village-level variable, while the income level of farmers belongs to the micro-individual-level variable. These two variables are at different observation levels and do not directly affect micro-farmers’ income levels, thus satisfying the exogeneity principle of the instrumental variable.

### Empirical results and analysis

This study used Stata 16.0 software to estimates the impact of farmers’ adoption behaviour of the rice–crayfish co-culture model on income. The results of the joint estimation of farmers’ adoption behaviour of the rice–crayfish co-culture model and income models are shown in Table 1. The third column in Table 1 is the estimation results of the factors affecting farmers’ adoption behaviour. The fourth column is estimated results of the factors affecting the income of farmers adopting the rice–crayfish co-culture model. The fifth column is the estimated results affecting the income of farmers who have not adopted the rice–crayfish co-culture model.

**Table 1:** Simultaneous estimation of adoption behaviour and the income effect of the rice–crayfish co-culture model.

Variable name		Select equation		Income effect			
				Adopters		Non-adopters	
		Coeff	Std. Err	Coeff	Std. Err	Coeff	Std. Err
Individual characteristics	Gender	0.145	0.134	0.179	0.146	0.131	0.669
	Age	−0.028^***^	0.003	−0.203^***^	0.025	−0.063^***^	0.025
	Health status	0.145^***^	0.005	0.128^***^	0.027	0.138	0.162
Family characteristics	Ratio of elderly	−0.013	0.014	−0.511^*^	0.290	−0.049	0.047
	Relationship network	0.106	0.065	0.235^***^	0.032	0.841^***^	0.222
	Years of farming	0.026^***^	0.006	−0.011	0.018	−0.028	0.019
Operational characteristics	Cultivated land area	0.011^***^	0.002	0.096^***^	0.008	0.047^***^	0.004
	Soil fertility	0.027^*^	0.014	0.032	0.021	0.063	0.078
	Number of arable land	−0.069^***^	0.019	−0.423^***^	0.118	−0.158^**^	0.069
	Farmland distance	−0.210	0.144	−0.102^**^	0.043	−0.700^***^	0.026
Other characteristics	Eco-consciousness	0.030^*^	0.017	0.036^**^	0.015	0.045^**^	0.017
	Loan situation	−0.042	0.156	0.603	0.466	0.014	0.031
	Outsourcing service	−0.013^***^	0.000	−0.015^***^	0.000	−0.003	0.006
	Internet usage	0.085	0.156	0.241^***^	0.055	0.104^***^	0.027
Village characteristics	Village terrain	−0.441^**^	0.202	−1.067^*^	0.625	−0.475	0.372
	Village irrigation	0.008^**^	0.004	0.043^**^	0.021	0.019	0.014
	Distance from town	−0.018^**^	0.008	−0.131^**^	0.051	−0.030	0.041
Instrumental variable	Neighbourhood adoption behaviour	0.134^***^	0.047	–	–	–	–
	Constant term	0.960^**^	0.392	0.650	0.491	0.465^**^	0.186
	$ln\sigma_{\mu a}$	–	–	1.840^***^	0.027	–	–
	$\rho_{\mu a}$	–	–	0.457^***^	0.139	–	–
	$ln\sigma_{\mu n}$	–	–	–	–	1.560^***^	0.039
	$\rho_{\mu n}$	–	–	–	–	−0.243^***^	0.067
	*LR*	18.330^**^	–	–	–	–	–

Notes: *, **, *** represent the significance levels of 10, 5, and 1 percent, respectively. 1 mu = 667m2.

The correlation coefficients $\rho_{\mu a}$ and $\;\rho_{\mu n}$ represent the correlation between the behaviour model and the income of the adopters and the income error term of the non- adopters. If the two correlation coefficient estimates are significant, it indicates that the sample has a self-selection problem. Table 2 indicates that the two correlation coefficients are significant at the 1% level, that is, the adopters and non-adopter groups are not randomly generated, and there is a certain level of self-selection.

**Table 2:** Descriptive statistics.

Variables	Measurements	Adopters	Non- adopters	Differences
		Mean	Mean	
Adoption behaviour	Did the respondents adopt the rice–crayfish co-culture model? 1 = Yes; 0 = No	1.000	0.000	–
Total income	Logarithm of household per capita net income (thousand yuan/year)	2.484	2.270	0.214^***^
Farm income	Logarithm of per capita farm net income of households (thousand yuan/year)	1.889	1.181	0.708^***^
Off-farm income	Logarithm of off-farm net income per capita of households (thousand yuan/year)	2.070	2.163	−0.093
Gender	Respondent’s gender. 1 = male; 0 = female	0.983	0.964	0.019^**^
Age	Respondent’s actual age (years)	54.958	59.658	−4.700^***^
Health status	Is the respondent in good physical condition? 1 = strongly disagree, 2 = disagree, 3 = average, 4 = agree, 5 = strongly agree	4.260	4.003	0.257^***^
Ratio of elderly	Population aged 65 and over as a proportion of the total household population	0.230	0.259	−0.029
Relationship network	Logarithm of human relationship expenses (thousand yuan/year)	10.997	9.193	1.804^***^
Farming years	Engaged in agricultural production time (years)	10.694	28.387	−17.693^**^
Cultivated land area	The actual business area (1 mu = 667m^2^)	30.262	23.304	6.958^*^
Soil fertility	Is the soil fertility of arable land better? 1 = strongly disagree, 2 = disagree, 3 = average, 4 = agree, 5 = strongly agree	3.433	3.155	0.278^***^
Number of arable land	The number of larger blocks of arable land (blocks)	2.618	3.515	−0.897^*^
Farmland distance	Distance between the two furthest farmlands (km)	0.450	0.572	−0.122^**^
Eco-consciousness	Actively recycle pesticide bottles, fertilizer packaging bags, etc.? 1 = Yes; 0 = No	0.786	0.651	0.135
Loan situation	Are there loans for agricultural production? 1 = Yes; 0 = No	0.265	0.223	0.042
Outsourcing service	Average cost of outsourcing in production (yuan/mu)	217.530	113.510	104.020^**^
Internet usage	Using the internet (mobile internet)? 1 = Yes; 0 = No	0.826	0.670	0.156^***^
Village terrain	What is the main terrain of the village? 1 = plain; 2 = hill	1.124	1.143	−0.019
Village irrigation	Proportion of effective irrigation area of village ditches (%)	96.450	97.000	−0.550
Distance from town	Distance from the village committee to the township (km)	6.910	5.577	1.333^**^
Human capital	Number of labour: number of farmers (person)	1.925	1.839	0.086^***^
	Quality of labour: average education level of farmers (years)	7.650	7.015	0.635^***^
Sample size	–	743	336	–

Notes:

* ,

** ,

*** represent the significance levels of 10, 5, and 1 percent, respectively. 1 mu = 667m^2^. The note is the same for the table below.

### Analysis of the influencing factors of adoption behaviour and income effect of the rice–crayfish co-culture model

By analysing the income effect of the adoption and non-adoption of the rice–crayfish co-culture model, we find that there are differences in the impact of different explanatory variables on income as follows:

(1) Individual characteristics. Age has a significant negative impact on the total income of both the adopters and non-adopters, that is, as the age of farmers’ increases, their family income decreases. This indicates that whether the rice–crayfish co-culture model is adopted or not, the age of the labour force is a key factor that affects their income level. The deep-seated reason for this is that the physical fitness of the labour force declines with age, which reduces the level of their participation in labour productivity. Moreover, the aging labour force is in a state of inefficiency in terms of work efficiency and quality, resulting in a greatly reduced labour productivity, which affects income.
(2) Family characteristics. There are differences in the impact of the proportion of elderly on the total income of the adopters and non-adopters, which has a significant negative impact on the adopters, that is, an increase in the proportion of the elderly is not conducive to improving the income level of the adopters. This is because an increase in the proportion of the elderly will affect the allocation of labour resources in adopters. Adopting the rice–crayfish co-culture model requires farmers to invest a lot of time in operation and management, which implies higher requirements for the allocation of labour resources in adopters. Taking care of the elderly also disperses the labour resources of the adopers, making it impossible to carry out refined management, thereby negatively affecting the increase in income.
(3) Operational characteristics. The cultivated land area, number of arable land blocks and farmland distance have a significant impact on the total income of both the adopters and non-adopters. Among them, the effect of cultivated land area is positive. As a key element of agricultural production, arable land resources are the foundation for maintaining national food security and realising agricultural modernisation, and within a certain range, the expansion of the operation scale will help optimise the allocation of agricultural production resources, realise the increase of scale returns and promote the increase in farmers’ income. Furthermore, the number and distance of arable land reflect the problem of arable land fragmentation, which directly limits the output benefits of arable land and leads to serious production marginalisation, which is a key constraint to the high-quality development of China’s agriculture.

Comparing with the results of existing studies, it can be seen that the education level of family members and family size have a significant positive impact on farmers’ continuous participation in the integrated aquaculture-agriculture(IAA) value chain [15]. This result provides support for this study.

### The effect of adopting the rice–crayfish co-culture model on farmers’ income

The objective is to estimate the impact of the adoption of the rice–crayfish co-culture model on farmers’ income. Table 3 shows that after eliminating the bias caused by unobservable factors, adopting the rice–crayfish co-culture model has a positive treatment effect on the total income of farmers and is significant at the 1% level. According to the ATT estimation results, for farmers who adopted the rice–crayfish co-culture model, if they had not adopted it, their total income would have dropped by 22.423%. The ATU reveals that for farmers who did not adopt the rice–crayfish co-culture model, if they had adopted it, their total income would have increased by 28.063%. Therefore, the adoption of the rice–crayfish co-culture model is helpful for the growth of the income of farmers, and H1 is preliminarily verified. The more general meaning of this conclusion is that the rice–crayfish co-culture model is not only a typical example of comprehensive rice farming in China but also an innovative application of the “double water and double green” model. It is mainly supported by the theory of two types of agriculture, adheres to the basic principle of adapting to local conditions, leads to the supply of green agricultural products and its effect of stabilising grain and increasing income is obvious.

**Table 3:** The average treatment effect of adopting the rice–crayfish co-culture model on farmers’ income.

	Type	Adopters	Non-adopters	ATT	ATU	Change (%)
Total income	Rice–crayfish co-culture model is adopted	2.484	1.927	0.557^***^	–	22.423
	Rice–crayfish co-culture model is not adopted	2.341	1.828	–	0.513^***^	28.063

Notes: *** represent the significance levels of 10, 5, and 1 percent, respectively.

Literature studies related to this study indicate that economic assessments have confirmed gross profit as the primary motivation for farmers to transition from traditional rice monoculture (RM) to integrated rice-crayfish farming (IRCF). The latter practice has been shown to achieve a 297% higher economic benefit compared to the former, highlighting its significant financial advantages in agricultural production systems [28]. However, this literature only evaluated the gross profit of the rice–crayfish co-culture model and did not systematically measure the income effect of farmers’ adoption of the rice–crayfish co-culture model.

### The impact of adopting the rice–crayfish co-culture model on different types of income of farmers

According to theoretical analysis, the adoption of the rice–crayfish co-culture model may not only increase farmers’ income but also impact different types of farmers’ income; the specific impact needs to be further investigated. Table 4 indicates that without considering the interference of other factors, the counterfactual estimation reveals that if farmers who have adopted the rice–crayfish co-culture model had not adopted it, their farm income would have dropped by 35.487%, and their non-farm income would have increased by 25.371%. Therefore, although the adoption of the rice–crayfish co-culture model helps increase farm income, it is not conducive to non-farm income. The increasing effect on farm income is greater than the inhibitory effect on non-farm income, which indicates that the adoption of the rice–crayfish co-culture model has different effects on different types of farmers’ income, supporting H1.

**Table 4:** The impact of adopting the rice–crayfish co-culture model on different types of income of farmers.

	Type	Adopters	Non-adopters	ATT	ATU	Change (%)
Farm income	Rice–crayfish co-culture model is adopted	1.888	1.218	0.670^***^	–	35.487
	Rice–crayfish co-culture model is not adopted	1.700	1.180	–	0.520^***^	44.068
Non-farm income	Rice–crayfish co-culture model is adopted	1.609	2.156	−0.547^***^	–	−25.371
	Rice–crayfish co-culture model is not adopted	2.077	2.175	–	−0.098^***^	−4.506

Notes: *** represent the significance levels of 10, 5, and 1 percent, respectively.

Literature studies related to this study indicate that rice–crayfish co-culture model has a robust positive and significant impact on farm household welfare measured by household annual income, farm income [21]. Nevertheless, this study failed to disaggregate farmers’ income components, nor did it clarify the mechanism behind the impact of adopting the rice–crayfish co-culture model on farmers’ income.

### Mechanism test of the impact of adopting the rice–crayfish co-culture model on farmers’ income

The theoretical analysis reveals that the adoption of the rice–crayfish co-culture model mainly affects the total income of farmers through human capital, which is embodied in two aspects–labour quantity and labour quality. Therefore, the mechanism of adopting the rice–crayfish co-culture model on the total income of farmers is tested using the mediation effect model. Theoretically, if the mediating effect of human capital is significant, the adoption of the rice–crayfish co-culture model affects the total income of farmers through human capital (see Table 5 for details).

**Table 5:** The impact mechanism of adopting the rice–crayfish co-culture model on farmers’ income.

	Regression (1)	Regression (2)	Regression (3)	Regression (4)	Regression (5)	Regression (6)
	Total income	Labour quantity	Total income	Total income	Labour quality	Total income
Adoption behaviour	0.381^***^	0.566^***^	0.281^***^	0.381^***^	0.341^**^	0.323^***^
	(0.079)	(0.157)	(0.051)	(0.079)	(0.157)	(0.150)
Labour quantity	–	–	0.053^**^	–	–	–
	–	–	(0.021)	–	–	–
Labour quality	–	–	–	–	–	0.161^***^
	–	–	–	–	–	(0.059)
Con-variables	Yes	Yes	Yes	Yes	Yes	Yes
R^2^	0.350	0.431	0.385	0.350	0.375	0.406

Notes: Robust standard errors are in brackets. The control variables are not presented due to space limitations

**, *** represent the significance levels of 10, 5, and 1 percent, respectively. Robust standard errors are in brackets. The control variables are not presented due to space limitations.

1. The mediating effect of the labour quantity. Regression (1) in Table 6 indicates that the adoption of the rice–crayfish co-culture model has a significant direct effect on the total income of farmers. Regression (2) indicates that the adoption of the rice–crayfish co-culture model has a significant positive impact on the labour quantity. Regression (3) reveals that the estimated results of both variables are significant, that is, after controlling for adoption behaviour, the impact of the labour quantity on the total income of farmers is still significant. This indicates that the mediating effect of labour quantity exists, which is a partial mediating effect, and the proportion of the mediating effect to the total effect is 0.079. This implies that approximately 7.9% of the impact of the adoption of the rice–crayfish co-culture model on the total income of farmers in the research area is achieved through the intermediary effect of the labour quantity.
2. The mediating effect of labour quality. Regression (5) in Table 5 indicates that the adoption of the rice–crayfish co-culture model has a significant positive impact on labour quality. Regression (6) indicates that after controlling the adoption behaviour, the impact of labour quality on the total income of rural households is still significant. The mediating effect of labour quality is inferred by the mediation effect test method, which is still a partial mediation effect, and the mediation effect accounts for 0.14 of the proportion of the total effect. This means that approximately 14% of the impact of the adoption of the rice–crayfish co-culture model on the total income of farmers is achieved through the quality of labour.

**Table 6:** Robustness test1.

Type	Adopters	Non-adopters	ATT	ATU	Change (%)
Rice–crayfish co-culture model is adopted	1.366	0.768	0.598^***^	–	43.778
Rice–crayfish co-culture model is not adopted	1.117	0.776	–	0.351^***^	45.232

Notes: *** represent the significance levels of 10, 5, and 1 percent, respectively.

Literature studies related to this study indicate that, a 22-hectare field experiment found that the combination of rice-fish symbiosis and micro-nano bubbles (MNBs) increased rice yield by 26.8% and is expected to improve economic efficiency by 35% [47]. However, this study did not further explore the specific mechanisms by which the rice–crayfish co-culture model promotes income growth for farmers.

### Robustness test

To test the reliability of the results, the robustness test is mainly performed by replacing the dependent variable. Here, we replace the total income of farmers with household consumption expenditure (thousand yuan/year) because the income of farmers is the material basis of consumption, and an increase in income can encourage farmers to increase consumption to a certain extent, so consumption can reflect the income status of farmers. Table 6 reveals that regarding the ATT, for adopers, if they had not adopted, their living consumption expenditure would have decreased by 43.778%, whereas if non-adopters had adopted the rice–crayfish co-culture model, their living consumption expenditure would have increased by 45.232%. The results are consistent with the benchmark regression results, indicating that the results in this paper are robust.

To further test the reliability of the research results, a second round of robustness testing was conducted using a different research method. This was achieved using two-stage least squares (2SLS). In the regression (1), the impact of the instrumental variable on the endogenous variable was examined. The results showed that the instrumental variable significantly and positively influenced the endogenous variable at the 1% statistical significance level, indicating that the instrumental variable was correlated with the endogenous variable. Additionally, the weak instrumental variable test result was 310.36, far exceeding the critical value of 10 and indicating the absence of a weak instrumental variable issue. After addressing the endogeneity issue, the regression (2) showed that adopting the rice–crayfish co-culture model significantly and positively affected farmers’ income at the 5% statistical significance level. These results are consistent with the baseline regression results and further confirm the reliability of the findings in this study(see Table 7 for details).

**Table 7:** Robustness test2.

Variable name	Regression (1)		Regression (2)	
	COE	Standard error	COE	Standard error
Adoption behaviour	–	–	4.359^**^	1.995
Instrumental variable	0.809^***^	0.046	–	–
Control variables	Yes	–	Yes	–
Constant term	0.203	0.152	9.375^*^	5.446
Weak tool testing	310.36	–	–	–
R^2^	0.310	–	0.100	–
Sample size	1079			

Notes: Please refer to the da ta.xls document in the Supporting Information for specific data sources

*, **, *** represent the significance levels of 10, 5, and 1 percent, respectively.

## Conclusions and policy recommendations

We analyzed data from 1,058 farmers households collected from one major agricultural producing province in China. Using the endogenous switching regression model (ESR) model and the mediation effect model, this study empirically analyzes the impact mechanism of adopting the rice–crayfish co-culture model on farmers’ income. The result reveals that: 1) Income growth amplification: Adopting the rice–crayfish co-culture model has a significant and quantifiable positive impact on farmers’ total income. Under the counterfactual framework, the total income of farmers who implemented the rice–crayfish co-culture model would have dropped by 22.423% if they had not done so. 2) Differential impact on income sources: The influence of adopting the rice–crayfish co-culture model on farm and non-farm income varies significantly, with a more substantial effect on farm income. This is because the fact that the rice–crayfish co-culture model directly transforms traditional single-crop rice farming into a more diversified and integrated agricultural production system. 3) Dual pathways of income increase: Adopting the rice–crayfish co-culture model directly boosts farmers’ income through increased rice and crayfish production and sales, it also indirectly raises income levels by enhancing human capital.

Based on the above conclusions, the main policy recommendations are as follows:

First, further unleash the potential of the rice–crayfish co-culture model to increase farmers’ incomes. On the one hand, the government can establish special funds to subsidize farmers who adopt the rice–crayfish co-culture model, reducing their initial costs and encouraging adoption, this will promote income growth among farmers. On the other hand, for farmers engaged in the production, processing, and sales of agricultural products related to the rice–crayfish co-culture model, agricultural special product taxes and value-added taxes should be appropriately reduced, this will lower operational costs, improve product market competitiveness, and enable more farmers to benefit from the model and achieve stable income growth.

Second, local governments should establish an industrial chain support system centered on the rice–crayfish co-culture model to increase agricultural profitability. On the one hand, they should cultivate a regional public brand by encouraging scattered farmers to form industrial consortiums. These consortiums should formulate unified management standards for the rice–crayfish co-culture model, improving operational standardization among farmers and thereby increasing their agricultural income. On the other hand, develop a rice–crayfish co-culture manager, which integrates intelligent sensors to monitor key indicators such as water temperature and dissolved oxygen in real time. Combined with an agricultural calendar, the manager will provide personalized management plans, and offer vouchers for agricultural inputs to farmers who adopt its recommendations, this will ultimately achieve the goal of increasing farmers’ income.

Third, when promoting rural income augmentation through the rice–crayfish symbiotic cultivation model, the pivotal role of human capital endowment warrants due consideration. For practitioners of the rice–crayfish co-culture system, targeted technical training programs should be implemented by governmental agencies. These programs aim to improve farmers’ proficiency in this cultivation model, enhancing the synergistic effect between the rice–crayfish co-culture system and human capital, ultimately boosting income growth. Regarding non-adopters, government bodies should engage in comprehensive dissemination efforts based on the principle of regional adaptation. By actively publicizing technical specifications, economic benefits, and ecological advantages of the rice–crayfish co-culture model, policymakers can effectively elevate farmers’ cognitive level and awareness of this innovative cultivation approach.

This study focuses on the practical adoption of the rice–crayfish co-culture model by micro-level farmers, using a combination of mathematical derivation and empirical analysis, the study explores the impact of adopting the rice–crayfish co-culture model on farmers’ income. The discussion reveals that:

First, as a typical practice in agricultural modernization, adopting the rice–crayfish co-culture model helps increase farmers’ income, with an income-increasing effect of 22.423%. Therefore, promoting the rice–crayfish co-culture model in the middle and lower reaches of China’s Yangtze River could achieve the goal of increasing farmers’ income. Notably, previous studies predominantly compared the revenue benefits of rice–crayfish co-culture against traditional monocropping systems [21]. In contrast, this study quantitatively analyzes the income effects of adopting the rice–crayfish co-culture model from the perspective of individual farming households, filling a crucial gap in the literature.

Second, the study reveals that human capital is a linchpin in farmers’ decision to adopt the rice–crayfish co-culture model. This discovery not only offers a theoretical framework for understanding the behavioral disparities among farmers but also serves as a cautionary note for policymakers. Neglecting human capital development while promoting modern agricultural models risks perpetuating a “low-efficiency” cycle, as the “human capital deficit” undermines implementation effectiveness. Notably, previous studies predominantly center on the economic and ecological advantages of the rice–crayfish co-culture model [27,29]. However, there remains a significant gap in exploring how the rice–crayfish co-culture model drives farmer income growth, along with a need to elucidate the underlying mechanisms in greater detail.

Finally, the findings of this study reveal that the adoption of the rice–crayfish co-culture model primarily increases farmers’ agricultural income, while its impact on non-agricultural earnings is relatively modest. Empirical data show that without the rice–crayfish co-culture model, farmers practicing this integrated model would have experienced a 35.487% decline in agricultural income. Notably, prior research has primarily focused on the effects of the rice–crayfish co-culture model on overall household income, neglecting to disaggregate income sources and analyze their individual contributions.

## Supporting information

S1 File The data analysis process can be found in the do file.do document. (DO)

S2 File The raw data can be found in the da ta.xls document. (XLSX)
